# Targeting of the MYCN Protein with Small Molecule c-MYC Inhibitors

**DOI:** 10.1371/journal.pone.0097285

**Published:** 2014-05-23

**Authors:** Inga Müller, Karin Larsson, Anna Frenzel, Ganna Oliynyk, Hanna Zirath, Edward V. Prochownik, Nicholas J. Westwood, Marie Arsenian Henriksson

**Affiliations:** 1 Department of Microbiology, Tumor and Cell Biology, Karolinska Institutet, Stockholm, Sweden; 2 Section of Hematology/Oncology, Children's Hospital of Pittsburgh of UMPC, Pittsburgh, Pennsylvania, United States of America; 3 School of Chemistry and Biomedical Sciences Research Complex, University of St. Andrews and EaStCHEM, St. Andrews, Fife, Scotland, United Kingdom; University of Tuebingen, Germany

## Abstract

Members of the *MYC* family are the most frequently deregulated oncogenes in human cancer and are often correlated with aggressive disease and/or poorly differentiated tumors. Since patients with *MYCN*-amplified neuroblastoma have a poor prognosis, targeting MYCN using small molecule inhibitors could represent a promising therapeutic approach. We have previously demonstrated that the small molecule 10058-F4, known to bind to the c-MYC bHLHZip dimerization domain and inhibiting the c-MYC/MAX interaction, also interferes with the MYCN/MAX dimerization *in vitro* and imparts anti-tumorigenic effects in neuroblastoma tumor models with *MYCN* overexpression. Our previous work also revealed that MYCN-inhibition leads to mitochondrial dysfunction resulting in accumulation of lipid droplets in neuroblastoma cells. To expand our understanding of how small molecules interfere with MYCN, we have now analyzed the direct binding of 10058-F4, as well as three of its analogs; #474, #764 and 10058-F4(7RH), one metabolite C-*m/z* 232, and a structurally unrelated c-MYC inhibitor 10074-G5, to the bHLHZip domain of MYCN. We also assessed their ability to induce apoptosis, neurite outgrowth and lipid accumulation in neuroblastoma cells. Interestingly, all c-MYC binding molecules tested also bind MYCN as assayed by surface plasmon resonance. Using a proximity ligation assay, we found reduced interaction between MYCN and MAX after treatment with all molecules except for the 10058-F4 metabolite C-*m/z* 232 and the non-binder 10058-F4(7RH). Importantly, 10074-G5 and 10058-F4 were the most efficient in inducing neuronal differentiation and lipid accumulation in *MYCN*-amplified neuroblastoma cells. Together our data demonstrate MYCN-binding properties for a selection of small molecules, and provide functional information that could be of importance for future development of targeted therapies against *MYCN*-amplified neuroblastoma.

## Introduction

The MYC family members c-MYC, MYCN and L-MYC are transcription factors crucial for the regulation of normal cellular functions including proliferation, cell growth, differentiation, metabolism and apoptosis. However, the genes encoding these proteins are also the most frequently deregulated oncogenes in several types of human cancers [1], [2]. c-MYC and MYCN (hereafter MYC), exert their functions mainly through transcriptional modulation of their target genes. The C-terminal domain of MYC comprises a basic helix-loop-helix leucine zipper domain (bHLHZip), necessary for the dimerization with its partner MAX and for sequence-specific binding to DNA [3], while the N-terminal transactivation domain interacts with co-factors to regulate transcription [2]. There is a large overlap between the downstream targets of c-MYC and MYCN and insertion of the *mycn* gene into the *c-myc* locus can fully rescue the embryonic lethal phenotype of a c-*myc* knockout mouse [4]. However, in normal tissue the expression pattern of these two proteins differ significantly [5], [6]. In the developing embryo, *MYCN* is expressed in certain tissues including the central and peripheral nervous systems, lung and spleen, whereas in adults its expression is very low or absent. In contrast, *c-MYC* is expressed in all proliferating cells in adults [6]–[9].

In human tumors, oncogenic alterations in *MYC* are common and include point mutations that increase protein stability, gene amplification, gene translocation, and enhanced translation [1], [2]. *MYCN* is amplified in cancers such as neuroblastoma (NB), medulloblastoma, lung cancer and glioma [1], [10]–[12]. In NB, a pediatric cancer of the sympathetic nervous system, *MYCN*-amplification is strongly correlated with poor prognosis and advanced tumor stage, and these tumors are often resistant to multimodal therapy [11], [12]. MYC is therefore an attractive target for cancer therapy [13], [14]. It has been shown that downregulation of MYC leads to cancer cell growth arrest, senescence, enhanced apoptosis, differentiation and/or tumor regression in mouse models of human cancer [15]. Importantly, even transient downregulation of MYC has been reported sufficient to diminish the tumor burden in animal models [15], and the effects of MYC inhibition on normal tissue has been shown to be well tolerated and reversible in adult mice [16]–[23]. Several groups have made efforts to target MYC at different levels, including its transcription, translation, hetero-dimerization with MAX, as well as targeting its direct or indirect downstream targets [10], [14], [24]–[26]. A number of small molecular compounds inhibiting c-MYC-MAX dimerization have been identified [27], [28] and among them 10058-F4 is by far the most studied. Biophysical experiments have shown that it interacts with the C-terminal bHLHZip region of c-MYC [28]–[32]. A fluorescence polarization assay was used to determine the affinity as well as to identify the binding site of 10058-F4 on c-MYC using different deletions, point mutations and synthetic peptides [30]. NMR measurements confirmed that 10058-F4 binds to amino acid residues 402–412 in the bHLHZip domain of c-MYC [30]. Furthermore, metadynamic molecular simulations and an ion mobility mass spectrometry using a peptide corresponding to the identified binding site, indicated that the compound binds to an inactive, disordered conformation of c-MYC [31], [32]. Together these studies suggest that 10058-F4 inhibits the function of c-MYC in a direct manner by preventing c-MYC/MAX hetero-dimerization. Importantly, several reports have shown that 10058-F4 affects c-MYC expression and induces cell cycle arrest, inhibits cell growth, promotes apoptosis and confers chemo-sensitivity in a c-MYC specific manner in various cancer cell types [28], [33]–[35]. In addition, treatment of acute myeloid leukemia (AML) cells with 10058-F4 leads to myeloid differentiation [33]. The effect of 10058-F4 treatment *in vivo* has been investigated in xenograft models of prostate cancer but no significant antitumor activity could be observed, probably due to its rapid clearance and low potency [36]. In contrast, we have recently demonstrated anti-tumorigenic effects of 10058-F4 in two tumor models of *MYCN*-amplified neuroblastoma, suggesting that direct MYC inhibition using a small molecule is achievable *in vivo* [36].

**Figure 1:**
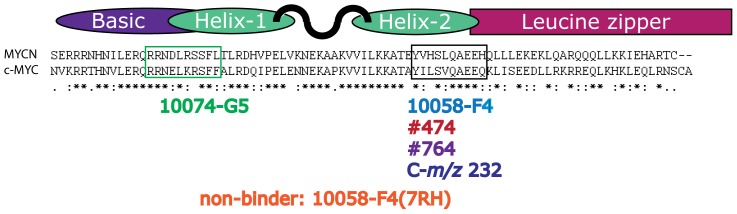
Alignment of the bHLHZip domains of c-MYC and MYCN. The amino acids in the bHLHZip domains of human c-MYC and human MYCN were aligned, while the secondary structure is indicated above. Identical amino acid are denoted (*), conserved substitution (:) and semi-conserved substitution (.). The two binding sites for the specified compounds as reported by Hammoudeh *et al* is indicated by a colored square [38]. Each small molecule is positioned under their reported or assumed binding site [38], [39]. For the 10058-F4 analogs #474 and #764 as well as its potential metabolite C-*m/z* 232 the binding sites have not been determined experimentally [39]. Through the similarity of their chemical structure to 10058-F4, it has been assumed that these compounds bind to the same site as indicated.

The structurally unrelated small molecule 10074-G5 was identified simultaneously as 10058-F4 as another substance that inhibits the c-MYC/MAX interaction. This molecule also decreased c-MYC protein levels and inhibited cell growth [28], but failed to show any antitumor activity in a xenograft model using a Burkitt's lymphoma cell line [37]. The cognate binding site for 10074-G5 on c-MYC was found to be distinct from that of 10058-F4, spanning amino acid residues 363–381 [30], [38] (see Figure 1). Both molecules were found to bind independently of each other, and probably induce only local conformation changes in the bHLHZip domain of c-MYC preventing its interaction with MAX [30], [38]. In order to identify more potent compounds, several analogs of 10058-F4 have been synthesized, some of which, including #015, #474 and #764, exhibited improved growth inhibition of c-MYC expressing cells [39].

Since c-MYC and MYCN share structural similarity in the bHLHZip domain we hypothesized that 10058-F4 also targets MYCN. We have previously shown that this compound interferes with the MYCN/MAX interaction leading to cell cycle arrest, apoptosis, and neuronal differentiation in *MYCN*-overexpressing NB cell lines [40]. In addition, using 10058-F4 as a tool, we found that inhibition of MYCN results in mitochondrial dysfunction leading to lipid accumulation. Importantly, 10058-F4 treatment furthermore increased the survival of TH-*MYCN* transgenic mice and showed anti-tumor effects in established aggressive NB xenografts [40].

Here, we determined the direct binding of 10058-F4 and additional selected c-MYC-targeting compounds to MYCN by surface plasmon resonance (SPR) (see Figure S1 for the structures of the compounds used). We found that all molecules previously reported to bind to c-MYC also bound to MYCN. Treatment with the small molecules furthermore interfered with the MYCN/MAX interaction and caused protein degradation, apoptosis, differentiation and lipid formation to different extents in *MYCN*-amplified NB cells.

## Results

### Folding of the human bHLHZip domain of MYCN and c-MYC

The bHLHZip domains of the MYC proteins are intrinsically disordered with little secondary structure without binding to MAX [41]. In order to verify the folding of the protein domains following purification under denaturing conditions and refolding, circular dichroism (CD) was performed. Comparison of the CD spectra of the purified and refolded bHLHZip domains with a model spectrum of a α-helical protein, indicated the presence of some α-helical structure in both c-MYC and MYCN (Figure S2). Analyses of the obtained spectra using the CONTIN, SELCON and the K2D algorithms [41]–[47] predicted the unstructured content to be very similar in both proteins, approximately 58% for c-MYC and 55% for MYCN. The α-helical structure was predicted to be 26% for c-MYC whereas MYCN had a slightly higher α-helical content, 33%. These values are in agreement with the 27% α-helical content documented for the v-MYC bHLHZip [48]. We found approximately 16% β-sheets in c-MYC and 13% in MYCN, respectively (Table S1). These results are indicative of two incompletely folded protein domains with some α-helical structure, as previously reported for the purified bHLHZip of c-MYC [30], [31]. Since the secondary structure content of the bHLHZip domains confirmed results from previous reports, we next performed surface plasmon resonance (SPR) experiments in order to examine the binding of the selected compounds to the purified bHLHZip domains of both proteins.

### Binding of small molecules to the bHLHZip of MYCN and c-MYC

The protein sequences of the human c-MYC and MYCN bHLHZip domains share 56% similarity (Figure 1). Given the highly structural and functional similarity of the C-terminal regions of c-MYC and MYCN it is very likely that molecules binding to c-MYC also bind to MYCN. In addition to 10058-F4 we included three of its structural analogs (Figure 1; Figure S1) in our study. The analogs #474 and #764 have previously been shown to have greater potency compared to the parent compound as judged by enhanced growth inhibition of c-MYC expressing leukemic cells [39]. In contrast, the analog 10058-F47RH (referred to as 7RH throughout) was found to be inactive in a c-MYC binding assay [39], and was thus included as a negative control. We also tested the binding to MYCN of a predicted *in vivo* metabolite of 10058-F4, C-*m/z* 232, in order to examine if the modified molecule still possesses some of the capacities of 10058-F4 [36]. Furthermore we included the structurally unrelated compound 10074-G5, previously shown to bind to c-MYC, in order to test the conservation of binding to a second site in the bHLHZip domain of MYC [28], [30], [38] (Figure 1).

**Figure 2:**
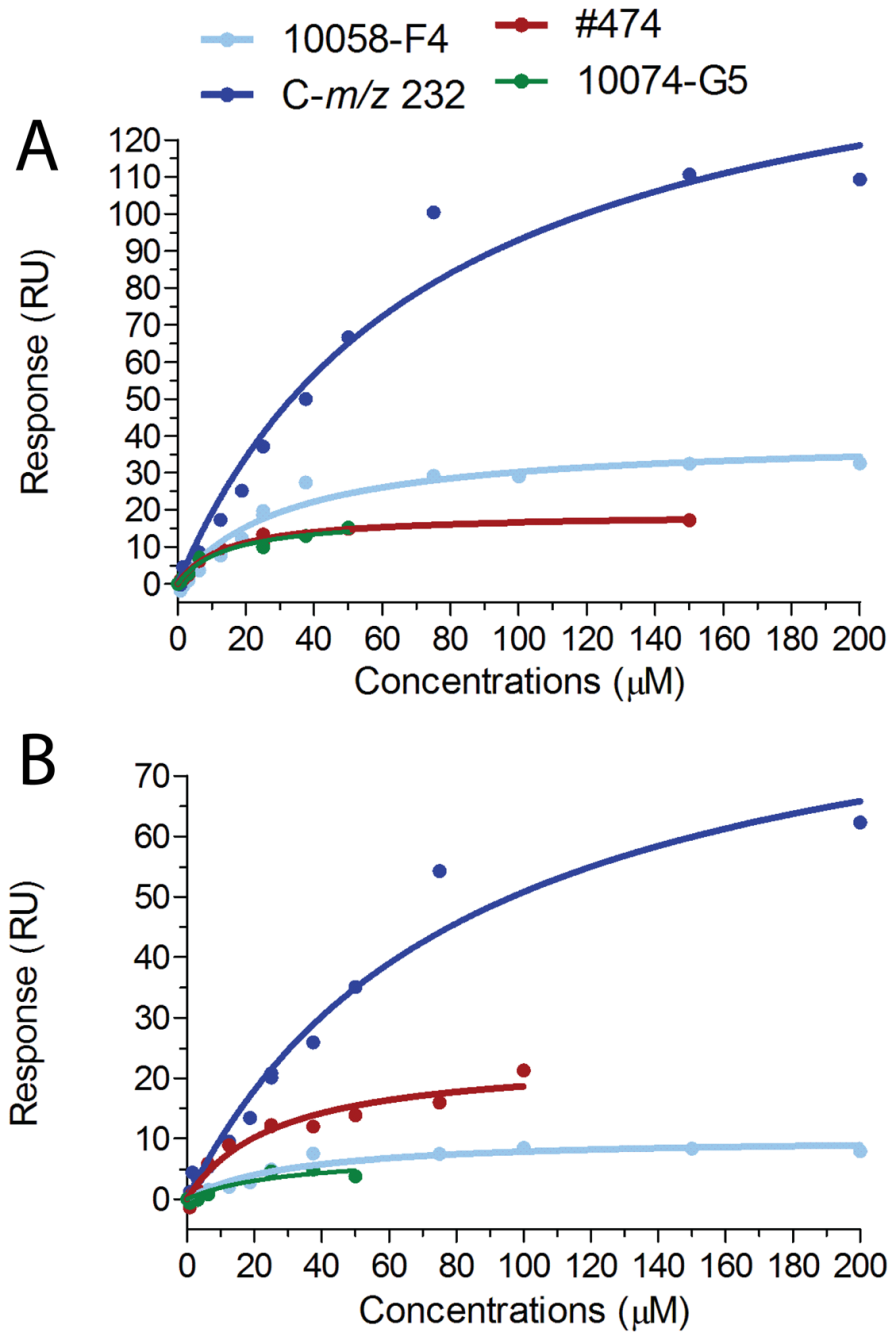
Equilibrium binding response for compounds binding to the bHLHZip of c-MYC and MYCN. Equilibrium binding response determined by Surface Plasmon Resonance of small molecules to c-MYC (A) or MYCN (B) as a function of the concentration as indicated. The concentrations were plotted versus the response units after solvent correction and subtraction from the reference surface. The plots are from one representative experiment. For each molecule the experiments were performed at least with two different immobilizations followed by at least three independent experiments.

For all SPR binding measurements the compounds were injected at increasing concentrations. After protein immobilization on the CM5 chip surface most of the c-MYC protein appeared to be active, since the expected maximal response (R_max_, the binding signal at saturation) was reached after injection of increasing concentrations of 10058-F4 (Figure S3). However for MYCN, only one fourth of the theoretical R_max_ was reached, indicating that not all protein molecules were able to bind to the analytes after immobilization (Figure S3). However, despite some of the MYCN protein being inactive, increased binding of the molecules was still detected in a dose-dependent manner and K_D_ values could be calculated for most of the compounds (Table 1, Figure 2 and Figure S3). Surprisingly, the obtained R_max_ values for C-*m/z* 232 to both c-MYC and MYCN were twice as high as those for 10058-F4, and double those of the theoretical R_max_ value for a single site binding to c-MYC, thus suggesting a possible second binding site. The analog #764 as well as 10074-G5 showed especially poor solubility in aqueous buffers and could not be analyzed at concentrations above 50 µM. Hence the R_max_ for c-MYC and MYCN could not be obtained for these molecules. Some unspecific binding, which was visible in the sensorgrams by a continuous, slightly upward trend of the curves, especially at higher concentrations, was detected for 10058-F4, C-*m/z*-232 and #474 (Figure S3), probably reflecting weak non-specific binding to other sites in c-MYC [30], [32] and MYCN, respectively. As expected, the 10058-F4 analog 7RH previously reported not to bind to c-MYC [39], was also a non-binder in our assays and was thus regarded as a negative control (Figure S3).

**Table 1:** K_D_ values of small molecules binding to the bHLHZip of c-MYC and MYCN.

Compound	c-MYC	MYCN
	K_D_ (µM)^a^	K_D_ (µM)^a^
10058-F4	39.7 (±8.1)	41.9 (±10.6)
C-*m/z* 232	82.3 (±5.5)	87.1 (±4.9)
#474	16.6 (±1.4)	26.9 (±4.9)
10074-G5	31.7 (±24.9)	19.2 (±11.5)

a KD values are shown as mean ± SD

In order to determine the affinity of the compounds to c-MYC and MYCN, the response for each small molecule was plotted against the analyte (compound) concentration. The K_D_ was defined as the concentration of analyte at 50% of the experimental maximal Response (R_max_). Approximate K_D_-values could be determined for all compounds except for #764. Similar affinities were determined for binding of 10058-F4 to both c-MYC and MYCN, with a mean K_D_ value of 39.7±8.1 µM and 41.9±10.6 µM, respectively (Table 1 and Figure 2). The affinities determined for the potential metabolite, C-*m/z* 232, were the lowest of all the molecules tested with a mean K_D_ of 82.3±5.5 µM for c-MYC and a mean K_D_ of 87.1±4.9 µM for MYCN. Compared to 10058-F4 the analog #474 analog showed an enhanced affinity both to c-MYC (mean K_D_ value of 16.6±1.4 µM) and to MYCN (mean K_D_ = 26.9±8.1 µM). Importantly, we demonstrated binding of the structurally unrelated compound 10074-G5 to both MYC proteins, and determined the affinity to both MYCN (K_D_ = 19.2±11.5 µM) and to c-MYC (K_D_ = 31.7±24.9 µM). Although the sensorgrams for #764 indicated binding to both proteins (Figure S3), we were not able to acquire data reliable enough to calculate its K_D_. As expected, binding of 7RH could not be detected to either of the two MYC proteins, even at the highest concentrations used (Figure S3). In summary, all active c-MYC inhibitors tested were also found to bind directly to MYCN.

### Targeting the MYCN/MAX interaction in NB cells

**Figure 3:**
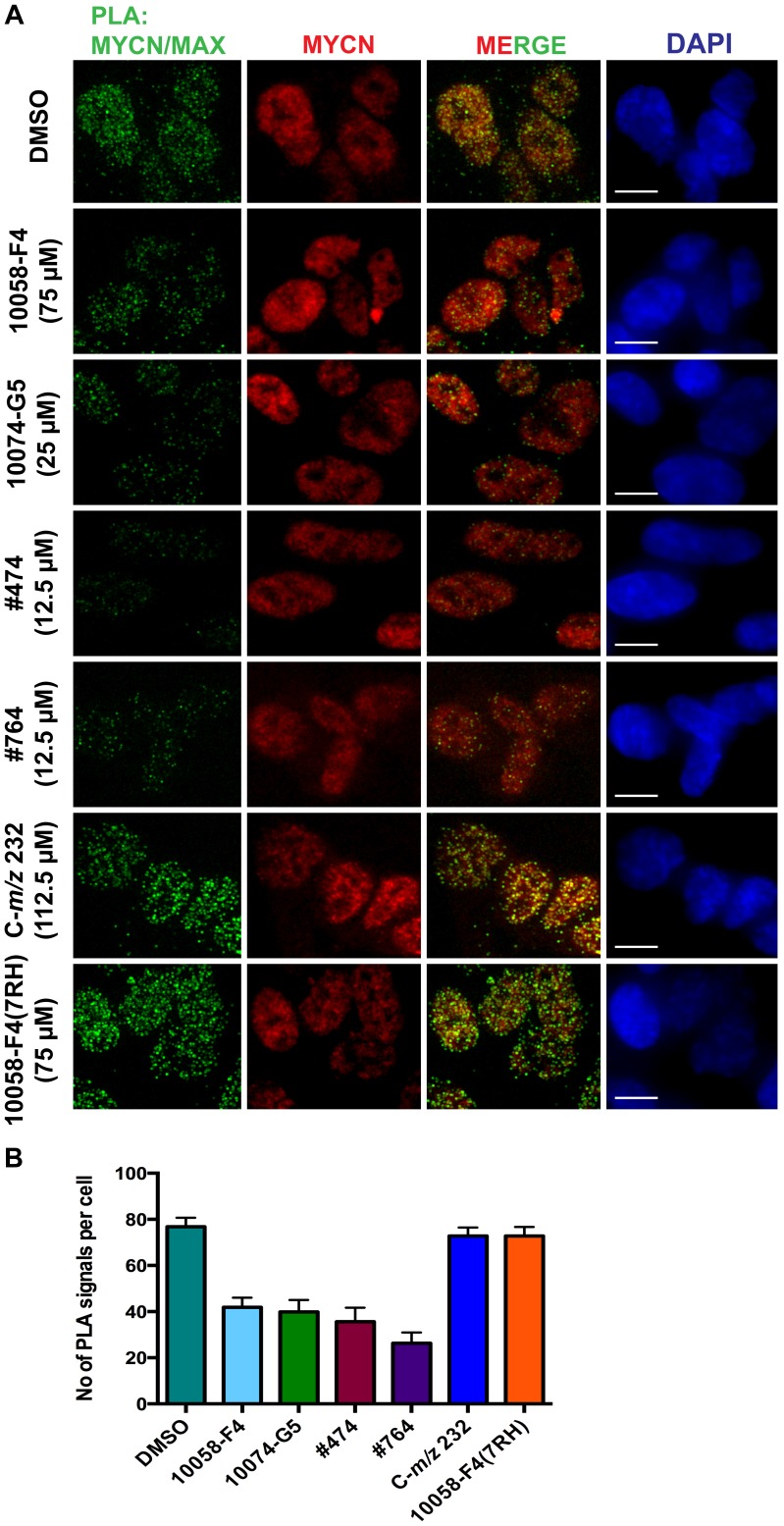
Reduction of MYCN/MAX interaction in NB cells after treatment with small molecules. A) Proximity ligation assay (PLA) for MYCN/MAX interaction after treatment of SMS-KCN69n cells with the different small molecules at the indicated concentrations for 6 hours. Green signals represent the proximity of the MYCN and MAX proteins, while the red signal shows the total MYCN signal in the cell. DNA is stained with DAPI. Scale bar: 10 µM. The pictures are representative from three independent experiments. B) Quantification of the PLA signals per cell after treatment with the different compounds. Error bars indicate standard error of the mean.

In our previous study we observed a reduction in MYCN/MAX interaction after treatment with 10058-F4 [40]. To investigate if the other small molecules also have an effect on MYCN/MAX dimerization we performed a proximity ligation assay (PLA) using *MYCN*-amplified SMS-KCN69n NB cells. Compared to DMSO treated cells, 10058-F4, its analogs #474 and #764 as well as the structurally unrelated compound 10074-G5 reduced the MYCN/MAX interaction following 6 hours treatment (Figure 3). In contrast, the metabolite C-*m/z* 232 and the non-binder 7RH did not affect MYCN/MAX dimer formation (Figure 3). No signal was detected in any of the experimental negative controls (Figure S4). In summary, all molecules that compared to 10058-F4 showed approximately similar or higher affinity to MYCN were also able to reduce the interactions between MYCN and MAX in NB cells.

### Decrease of MYCN protein levels after treatment with small molecules

**Figure 4:**
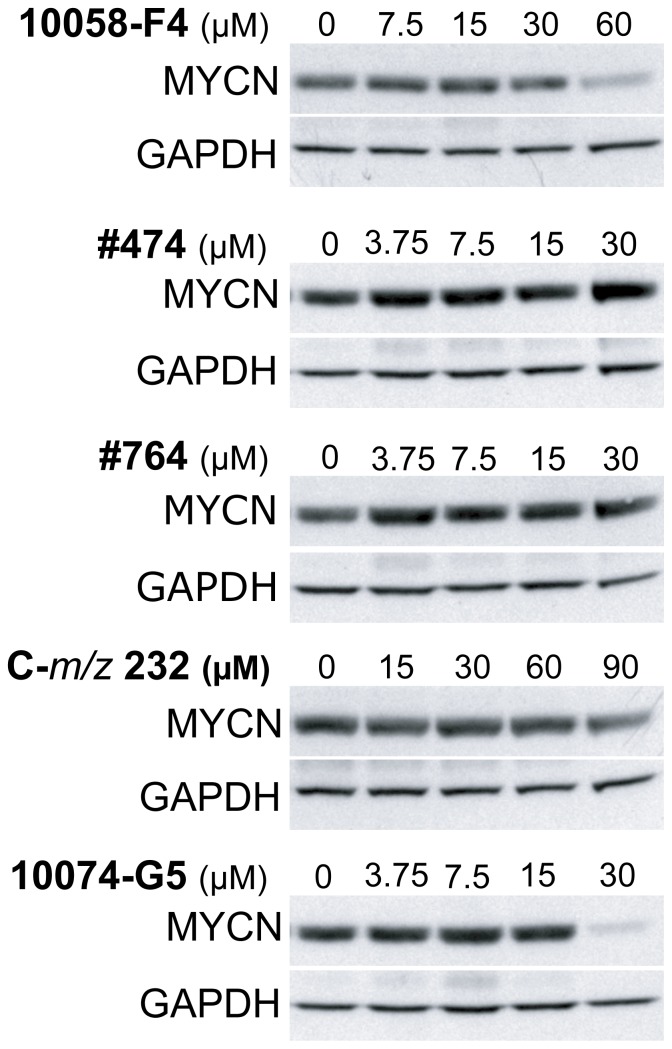
Reduction in MYCN protein levels in NB cells after treatment with small molecules. Western blot analysis of MYCN expression in SK-N-BE(2) cells treated for 48 hours with the indicated concentrations of 10058-F4, 10058-F4 metabolite C-*m/z* 232, 10058-F4 analogs #474 and #764 and 10074-G5. Membranes were probed with antibodies recognizing MYCN and GAPDH. The blots are representative from three independent experiments.

It has previously been reported that 10058-F4 treatment leads to down-regulation of c-MYC protein levels in *c-MYC* overexpressing cell lines and we observed similar results for MYCN levels in *MYCN*-amplified NB cells [33], [34], [40]. In order to determine the effect of the other compounds analyzed in this study on the MYCN protein levels, SK-N-BE(2) NB cells were treated with as a high concentration of each compound as possible without resulting in excessive cell death. While 10074-G5 decreased the MYCN protein levels at lower concentrations than 10058-F4, the putative metabolite C-*m/z* 232 showed only a slight reduction in the MYCN levels at high concentrations, and the 10058-F4 analogs #474 and #764 did not cause any changes in the MYCN protein levels at the concentrations which were possible to use without apoptosis induction (Figure 4). In summary, only 10058-F4 and 10074-G5 were able to efficiently decrease the MYCN protein levels.

### Effect of small-molecules on growth inhibition and apoptosis in MYCN-amplified cells

**Figure 5:**
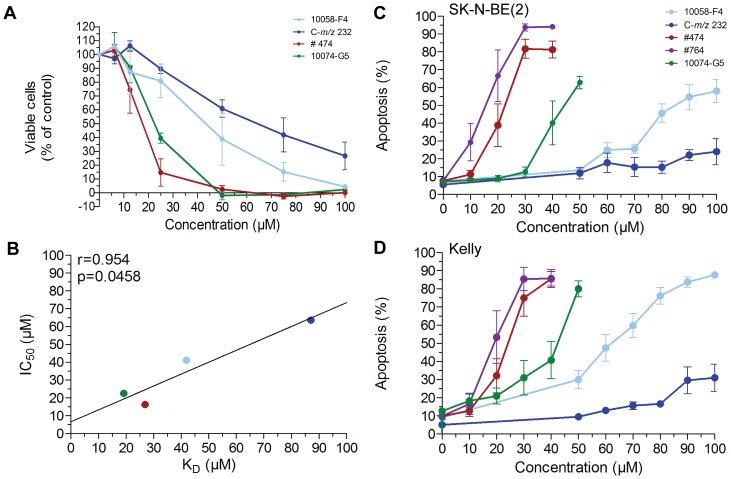
Growth inhibition and apoptosis induction in *MYCN* amplified cells. (A) The amount of viable of cells was determined using crystal violet assay following 48 hours treatment of Kelly cells with increasing concentration of the indicated compound. Data are shown as percent of control (DMSO) treated cells and represent the mean of three independent experiments. Error bars indicate standard deviation. (B) Pearson's correlation between the IC_50_ values in the growth inhibition assay (A, Table 2) and the K_D_ values for binding to MYCN (Table 1). (C–D) Quantification of apoptosis by propidium iodide staining for sub G1 DNA content of SK-N-BE(2) (C) and Kelly (D) cells. Data represent the means of at least three independent experiments. Error bars indicate standard deviation.

The small molecules 10058-F4, 10074-G5, as well as #474 inhibit cell proliferation and induce apoptosis in a variety of c-MYC expressing cells [28], [33], [34], [39], and we have shown that 10058-F4 induces apoptosis and inhibits cell growth in MYCN overexpressing NB cells [40]. Here we examined the effect on cell growth (and apoptosis) of the other compounds in *MYCN*-amplified Kelly NB cells. As shown in Figure 5A, all molecules tested inhibited cell growth in a concentration dependent manner. Compared to 10058-F4, the analog #474 and 10074-G5 were more potent whereas the metabolite C-*m/z* 232 caused less growth inhibition (Figure 5A). Importantly, the calculated IC_50_ values from the cell growth inhibition assays and the K_D_-values from the SPR experiment for binding to MYCN strongly correlated to each other (Figure 5B).

**Table 2:** IC_50_ values from the crystal violet assay in Kelly cells.

Compound	IC_50_ (µM)	95% Confidence Interval (µM)
10058-F4	41.1	33.9–49.7
C-*m/z* 232	63.6	55.2–73.3
#474	16.3	14.9–17.8
10074-G5	22.5	20.1–25.3

We next investigated the level of apoptosis induction in response to treatment with the molecules using two different MNA NB cell lines (Figure 5C, D). As previously shown, Kelly cells were more sensitive to 10058-F4 treatment than the SK-N-BE(2) cells [40]. This effect was also seen after treatment with 10074-G5, while the 10058-F4 analogs #474 and #764 were equally efficient in inducing apoptosis in the two cell lines (Figure 5C, D). The 10058-F4 analogs #474 and #764 as well as 10074-G5 were more potent than 10058-F4 in inducing cell death in both cell lines analyzed (Figures 5C, D). To verify the MYCN specificity in apoptosis induction, we next used Tet21N NB cells with doxycycline regulated MYCN expression. As shown previously [40], cells with low MYCN-levels were less sensitive to 10058-F4 treatment compared to cells with high MYCN protein levels. The MYCN dependent induction of apoptosis was also observed after treatment with 10074-G5, #474 and #764. In contrast, no apoptosis was observed after incubation with C-*m/z* 232 independently of MYCN status (Figure S5). As previously reported for c-MYC expressing cells [39], the effect of #764 on apoptosis induction was similar or slightly stronger than that of #474 in Tet21N cells (Figure 5C, D). In conclusion, the affinity of the molecules to MYCN correlated with their ability to inhibit cell growth and to selectively induce apoptosis in MYCN overexpressing NB cells (Figure S5).

### NB cells undergo neuronal differentiation in response to 10074-G5

**Figure 6:**
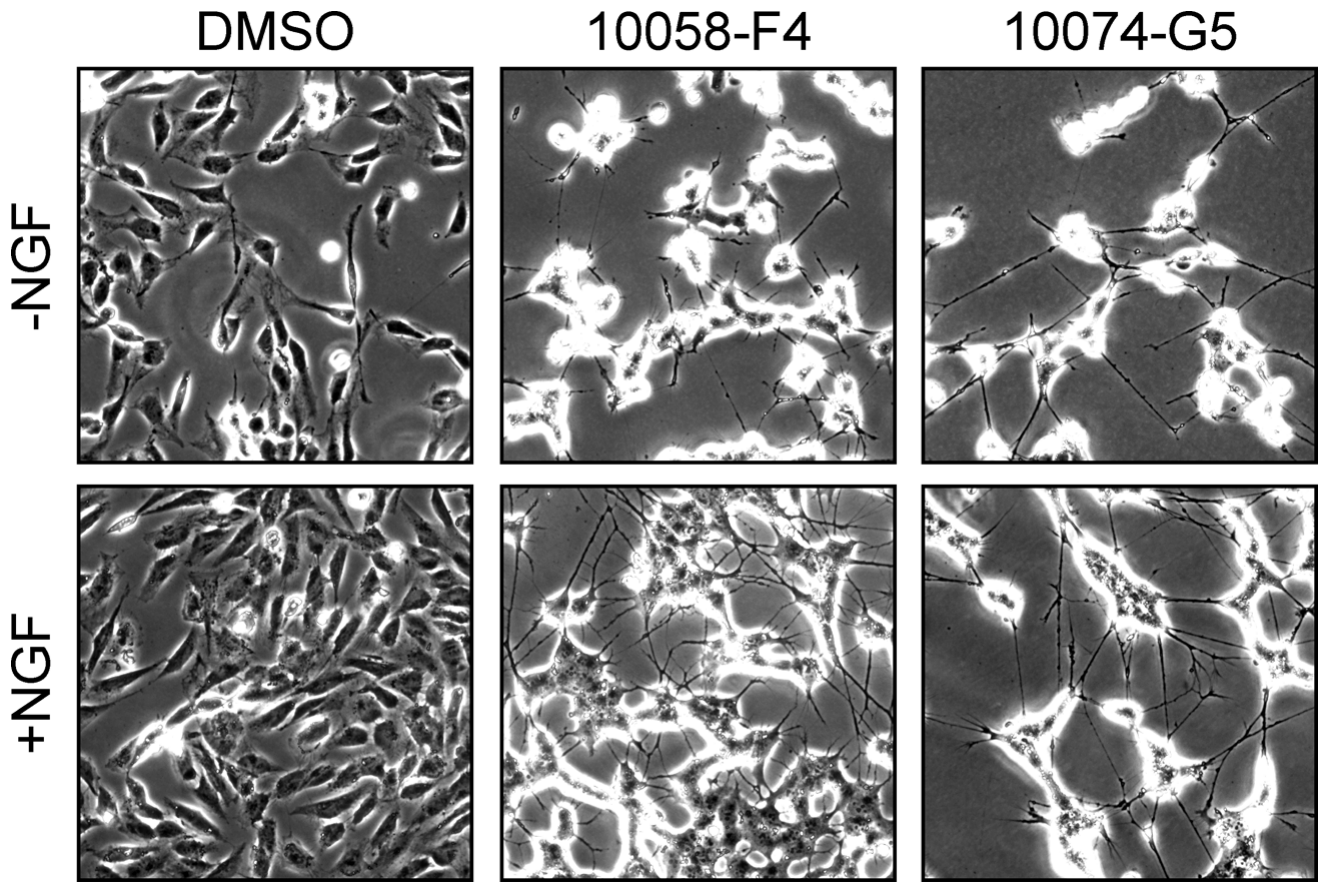
10074-G5 induces neuronal differentiation in NB cells. Morphological differentiation of SK-N-BE(2) cells in response to 15 days culture with 10058-F4 (60 µM) 10074-G5 (30 µM) or DMSO in the presence or absence of NGF (50 ng/ml). Phase contrast micrographs show representative pictures from one out of three independent experiments.

MYCN counteracts neuronal differentiation whereas inhibition of MYCN promotes differentiation of NB cells [49], [50]. We have previously shown that 10058-F4 stimulates NB differentiation, induces expression of the nerve growth factor (NGF) receptor TrkA and hence renders *MYCN*-amplified NB cells sensitive to NGF-mediated differentiation [40]. Similarly, treatment of SK-N-BE(2) cells with sub-lethal concentrations of 10074-G5 for 14 days also resulted in evident neurite outgrowth (Figure 6). As previously shown for 10058-F4 treatment, NGF potentiated the differentiated morphology of 10074-G5 treated cells by increasing both the number and the length of neurite protrusions (Figure 6). The same effect was observed after treatment with C-*m/z* 232, whereas incubation with neither #474 nor #764 resulted in morphological changes (Figure S6).

### Treatment of NB cells induces lipid accumulation

**Figure 7:**
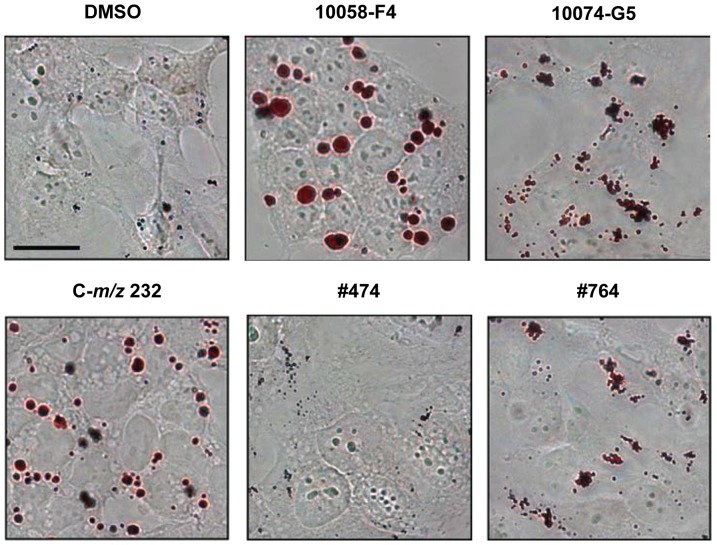
Lipid accumulation in *MYCN*-amplified NB cells. Oil red O staining of SK-N-BE(2) cells treated with 10058-F4 (60 µM), C-*m/z* 232 (90 µM), 10074-G5 (20 µM), #474 (10 µM), #764 (10 µM) or DMSO for 3 days. The scale bar corresponds to 20 µm. Pictures are representative from four independent experiments.

We have previously shown that 10058-F4 treatment as well as genetic down-regulation of MYCN using shRNA, leads to accumulation of lipid droplets in *MYCN*-amplified NB cells. We also demonstrated that 10058-F4 treatment results in down-regulation of proteins involved in β-oxidation of fatty acids as well as in the respiratory chain, and that these changes are likely to play a role in the observed lipid accumulation [40]. To explore the effect on lipid accumulation of the small molecules studied here cells were treated with sub-lethal concentrations of the compounds and stained with Oil Red O in order to detect lipids. As seen in Figure 7, treatment with C-*m/z* 232 at concentrations under 80 µM did not resulted in lipid accumulation, whereas at 90 µM the amount of lipid droplets was similar to after incubation with 10058-F4. Interestingly, 10074-G5 treatment resulted in smaller but more numerous lipid droplets compared to 10058-F4 and C-*m/z* 232. We also observed lipid accumulation, although less pronounced, after treatment with #474, whereas #764 led to only a minor increase in the amount of lipid droplets (Figure 7). Taken together, we have demonstrated that although the analyzed compounds all bind to MYCN, they induce lipid droplet accumulation to different extents.

## Discussion

To date several research groups have focused on developing compounds that target c-MYC for cancer therapy, whilst there have been only a few publications that have explored the possibility of targeting MYCN [27], [28], [39], [51], [52] The small molecule 10058-F4 has been extensively studied in the context of targeting c-MYC in cancer cells, although more information is needed regarding its true specificity [28], [30], [33], [34], [40]. Recently, we have shown that 10058-F4 also reduces MYCN/MAX interaction in addition to c-MYC/MAX and that it induces selective apoptosis and cell growth arrest in *MYCN*-amplified compared to non-*MYCN* amplified NB cells. Furthermore we demonstrated significantly delayed tumor growth in a NB xenograft and increased survival in a transgenic mouse model of NB [40]. These results are in contrast with a previous report that showed no significant antitumor activity of 10058-F4 in a xenograft model of prostate cancer [36]. The differences might be due to the targeting of MYCN as well as c-MYC by 10058-F4 in NB, as well as to a potential greater reliance of NB cells on MYCN for their survival.

Inspired by our earlier findings, we explored whether additional compounds shown to modulate c-MYC function could also bind MYCN and inhibit its function in NB cells. Here, extensive characterization of five compounds has been carried out including monitoring of their binding to MYCN as well as their effects in cellular assays used in our previous study.

### Comparison of 10058-F4 and three closely related structural analogs

Three of the selected compounds, known as #474, #764 and 7RH, are close structural analogs of 10058-F4 [39] (Figure S1). Both #474 and #764 have been previously reported to show improved interaction with c-MYC compared to 10058-F4, whereas 7RH does not bind to c-MYC and was therefore included as a negative control [39]. Our SPR analysis showed binding to both c-MYC and MYCN for all compounds tested except for the non-binder 7RH. The estimated K_D_ values showed a similar affinity of 10058-F4 to both proteins. The approximate affinities we could determine were higher than the originally reported K_D_-values [30], which might be due to the fact that in the fluorescence polarization assay the bHLHZip domain is free in solution, while in the SPR-based assay it is immobilized on the chip surface by amine coupling and thus physically constrained. However we were encouraged by the fact that 10058-F4 also bound to MYCN with an approximately equal affinity as to c-MYC.

10058-F4 is proposed to bind preferentially to Tyr402 and the hydrophobic region between residues 401 and 406 in c-MYC [32]. As shown in Figure 1, MYCN also contains a Tyr at the analogous position (residue 429) as well as a highly homologous hydrophobic region (residues 428–433). This suggests that 10058-F4 can adopt a similar binding mode for MYCN leading to the equivalent binding of 10058-F4 to c-MYC and MYCN. Whereas it proved difficult to assess the binding of #764 to c-MYC or MYCN due to lack of compound solubility, our studies indicated that #474 bound more strongly to c-MYC than 10058-F4. As expected, 7RH did not bind to c-MYC or to MYCN.

10058-F4, #474 and #764 were also able to decrease the interaction of MYCN and MAX as analyzed in a proximity ligation assay, whereas 7RH did not. Both #474 and #764 were more potent than 10058-F4 in the growth inhibition and apoptosis assays. Importantly in terms of assessing compound specificity, 10058-F4, #474 and #764 all induced apoptosis more effectively in MYCN expressing compared to non-MYCN expressing Tet21N cells. Surprisingly, the analogs #474 and #764 did not cause any decrease in the MYCN protein levels and probably therefore also did not induce neuronal differentiation. In addition, #764 only induced a few and very small lipid droplets compared to 10058-F4 and 10074-G5 whereas #474 treatment resulted in very little lipid accumulation. Taken together, these experiments suggest that the full biological effects of MYC inhibiting compounds are most apparent not only when the binding between MYCN and MAX is inhibited but when the levels of MYCN protein are reduced as well. Residual MYC activity may thus be sufficient to permit continued mitochondrial function, thus preventing the accumulation of neutral lipids.

### Analysis of the 10058-F4 metabolite C-*m/z* 232

In a previous study it was shown that C-*m/z* 232 is a major metabolite of 10058-F4 resulting from conversion of the thio-carbonyl group in 10058-F4 to the corresponding carbonyl group [36]. We compared 10058-F4 and C-*m/z* 232 across our series of assays to assess whether the activity observed for 10058-F4 in cells and in our animal model of NB could in fact be due to the presence of C-*m/z* 232. In general, C-*m/z* 232 retained some of the activities displayed by 10058-F4 but to a significantly reduced extent. More specifically, C-*m/z* 232, was able to bind to c-MYC and MYCN but with much lower affinity compared to 10058-F4, and whilst neuronal differentiation and the formation of lipid droplets were seen for cells treated with C-*m/z* 232, the phenotype was considerably less strong than that observed after 10058-F4 treatment.

Importantly, C-*m/z* 232 was not able to effect the MYCN/MAX interaction in the proximity ligation assay even at a concentration of 112.5 µM. In addition, treatment of cells with C-*m/z* 232 only slightly reduced the MYCN protein levels at a high concentration, and it only caused low levels of growth inhibition and apoptosis. Furthermore, we did not observe any induction of apoptosis in MYCN or non-MYCN expressing Tet21N cells. Taken together these results suggest that when 10058-F4 is used in cells or animal models, the contribution of the metabolite C-*m/z* 232 to the outcome of the experiment is only minor.

### Comparison of the 10058-F4 and 10074-G5 molecules

The 10074-G5 compound which is structurally unrelated to 10058-F4 has previously been reported to modulate c-MYC activity by preventing the c-MYC/MAX interaction. Importantly the regions of the bHLHZip domain of c-MYC that interact with these two molecules have been shown to be distinct [29], [36] (Figure 1). Here, we demonstrate that 10074-G5 also binds to MYCN. However due to the relatively large standard deviation associated with the data no more conclusions can be drawn other than that its affinity is approximate the same as that for 10058-F4.

In addition, a good correlation between the affinities of both 10058-F4 and 10074-G5 for MYCN and their inhibitory effects on *MYCN*-amplified NB cells was observed. Importantly, 10074-G5 reduced the MYCN/MAX interaction in NB cells as demonstrated by PLA. In general the observed activity of 10074-G5 across the wide range of cell-based assays was very robust, and consistent with its previously reported rapid intracellular accumulation during cell culture [53]. In fact, 10074-G5 mirrored the effects of 10058-F4 but was more effective at reducing MYCN protein levels and was more potent for both growth inhibition and apoptosis induction. The greater degree of apoptosis seen in MYCN expressing compared to non-expressing Tet21N cells suggested selectivity for MYCN. The 10074-G5 molecule also induced robust neuronal differentiation and accumulation of lipid droplets. Studies aimed at optimizing 10074-G5 for disruption of the c-MYC/MAX have been reported recently [52], [53] and overall our data suggest that 10074-G5 is at least as promising as 10058-F4 for targeting of MYCN. Potential concerns with regard to a drug discovery however, relate to the presence of a nitro functional group and the benzofurazan core structure of this molecule.

In conclusion, we have carried out a comparison of several small molecules previously shown to bind directly to the bHLHZip of c-MYC in the context of direct binding to MYCN and modulation of MYCN activity. We found that all c-MYC binding molecules analyzed also bind to the corresponding region of MYCN and results in inhibitory activity against NB cells *in vitro*. Importantly our work highlights 10074-G5 as being of potential interest as a starting point for the development of next-generation inhibitors of the MYCN/MAX interaction.

## Materials and Methods

### Subcloning of the bHLHZip domain of human c-MYC and human MYCN

The gene fragments comprising human *c-MYC* residues 353–437 and human *MYCN* residues 380–464 were obtained by PCR amplification of cDNA encoding human *c-MYC* and *MYCN*. The forward primers used were 5′GTTGTCATATGAATGTCAAGAGGCGAACACACA3′ and 5′GTTGTCATATGAGTGAGCGTCGCAGAAACCACA3′ as well as the reverse primers 5′CAGCTACGGAACTCTTGTGCGTAA3′ and 5′TTGAACACGCTCGGACTTGCTAG3′ for *c-MYC* and *MYCN*, respectively. The PCR fragments were cloned into the pET28a vector (Novagen) under the control of a T7 promotor and introducing an N-terminal hexa-histidine tag after digestion with NdeI and BamHI. The identity of the plasmids was verified by sequencing.

### Preparation of the bHLHZip c-MYC and bHLHZip MYCN protein domains

N-terminally His-tagged bHLHZip c-MYC (353–437) and bHLHZip MYCN (380–464) proteins were over-expressed in *E. coli BL21* (DE3) bacteria (Stratagene) at 37°C in LB media with kanamycin. Cultures were induced with Isopropyl β-D-1-thiogalactopyranoside (0,1 mg/ml) at an OD of 0.6 and harvested after 4 hours. After sonication of the proteins in 50 mM NaH_2_PO_4_, 100 mM NaCl and 10 mM Tris-HCl (pH = 8,0) for 6×30 seconds, the pellet was resuspended in 100 mM NaH_2_PO_4_, 10 mM Tris-HCl, 10 mM imidazole and 8 M urea (pH = 8,0) prior to additional sonication for 6×30 seconds. The proteins were then loaded onto a Ni-Sepahrose High Performance His-Trap column (GE Healthcare), washed with ten column volumes of 100 mM NaH_2_PO_4_, 10 mM Tris-HCl, 40 mM imidazole and 8 M urea (pH = 8,0) and finally eluted with two column volumes 100 mM NaH_2_PO_4_, 10 mM Tris-HCl, 500 mM imidazole and 8 M urea (pH = 8,0). Fractions without visible higher molecular mass contaminations, as judged by SDS-page, were pooled and refolded by dialysis in 50 mM NaH_2_PO_4_, 100 mM NaCl, 10 mM Tris-HCl (pH = 8,0), 5 mM EDTA, 2 mM DTT and decreasing concentrations of urea (6 M, 4 M, 2 M and 0 M). Finally the buffer was exchanged to either PBS (Hyclone) for use in circular dichroism measurements or 20 mM Na_2_HPO_4_*2H_2_O, 300 mM NaCl, 4 mM KH_2_PO_4_, 0,05% Surfactant P20 (GE Healthcare) and 5% DMSO (pH = 7,5) for use in Surface Plasmon Resonance analysis. Afterwards the proteins were concentrated up to a concentration of 5 mg/ml. Protein concentrations were determined by SDS-page comparing purified proteins to a standard curve of BSA loaded on the gel.

### Circular Dichroism

The wavelength scan were performed with a protein concentration of 1 mg/ml in PBS at 20°C in a 1 mm path-length cell with a JASCO J-810 spectrometer equipped with a thermoelectric temperature controller. The residual secondary structure of the bHLHZip of c-MYC and MYCN was estimated by means of spectral deconvolution with CONTIN, SELCON and K2D on the Dichroweb server using reference sets 4 and 7. Reported values represent the average result of CONTIN, SELCON and K2D algorithms in percent as well as the relative standard deviation.

### Surface Plasmon Resonance

Surface Plasmon Resonance (SPR) experiments were performed using a Biacore T200 (GE Healthcare) instrument at 25°C. The proteins were immobilized on a CM5 (GE Healthcare) chip through amine coupling using the amine coupling kit (GE Healthcare) resulting in immobilization levels between 1900–2100 RU. For each immobilization a new purified batch of protein was used. The samples were flowed over the surface with 30 µl/min for 60 seconds with a regeneration time of 600 seconds. The experiments were performed in the same batch of running buffer as used for dialysis. After each injection the flow delivery system was washed with 50% DMSO to avoid possible binding of molecules. To remove all remaining analyte the surface was regenerated with 10 mM NaOH. The obtained data was analyzed by the Biacore T200 Evaluation Software 2.0 (GE Healthcare). R_max_ is the binding signal at saturation. The K_D_-values were obtained from steady state fitting of equilibrium curves by plotting the response against the concentration of the analyte (compound). The K_D_ value is calculated as 50% of the experimental R_max_. The theoretical Rmax  =  (MW analyte/MW ligand) x immobilized ligand level on the chip (RU).

### Cell culture

Human *MYCN*-amplified neuroblastoma SK-N-BE(2) and SMS-KCN69n cells were grown in MEME∶F12-Ham medium (1∶1, v/v) supplemented with 10% FBS, 1% glutamine, 1% non-essential amino acids, and 1% penicillin/streptomycin. Human *MYCN*-amplified neuroblastoma Kelly cells were grown in RPMI 1640 medium supplemented with 10% FBS, 1% glutamine, and 1% penicillin/streptomycin. Tet21N cells derived from the human non-*MYCN* amplified NB cell line SH-EP, contain a repressible (Tet-Off) *MYCN* gene and were cultured as previously described [51]. Doxycycline was added as indicated to repress *MYCN* expression at a final concentration of 1 µg/ml.

### Reagents

10058-F4 (1RH) and 10074-G5 were purchased from Sigma. 10058-F4 (7RH) (# 5321418), #474 (# 6123474), #764 (# 6863764) and C-*m/z* 232 (# 5955535) were purchased from Chembridge. Propidium iodide and doxycycline were from Sigma.

### Proximity Ligation Assay (PLA)

40×10^3^ SMS-KCN69n cells were seeded on collagen coated coverslips and grown for 24 hours and treated for 6 hours the next day. After 6 hours the cells were washed in PBS and treated as previously described [40]. The PLA assay was performed after manufacturer's instructions with the Duolink kit (Olink, Uppsala, Sweden). The cells were incubated with primary antibody against MYCN (NCMII100, St Cruz Bio) 1∶500 and Max (C-17, St Cruz Bio) 1∶500 for 1 hour in a humidity chamber at 37°C, which were diluted in antibody diluent provided by the manufacturer. MYCN (1∶500) and FAS (A-20, St Cruz Bio) 1∶500 as well as MAX (1∶500) and GFP (C-2, St Cruz Bio) 1∶500 antibodies were used in combination in order to test for any unspecific binding of the secondary antibodies or unspecific signals. All of the following procedures were performed according to manufacturer's instructions. During the amplification step an additional secondary antibody (Alexa fluor 568, goat anti-mouse; Invitrogen) was added 1∶1000 to the amplification solution to counterstain for MYCN protein.

### Microscopy platform

The confocal images of the cells were acquired at 63× magnification using a motorized axiovert fluorescence microscope 200 M LCI (Zeiss GmBH, Göttingen, Germany) including a CSU10 Yokogawa head; a multilens/multipinhole array aperture (Yokogawa, Japan), an ORCA ER cold CCD camera; detector array 1344×1024px (Hammamatsu, Japan) and two continuous wave diode-pumped solid-state (DPSS) lasers for fluorescence excitation (Cobolt, Sweden); Cobolt Calypso 491 nm DPSS (excitation of green PLA fluorophore) and Cobolt Jive 561 nm DPSS (excitation of Alexa Fluor 568). Emission filters 525/50 and 607/45 were used respectively. Open lab software (Perkin Elmer, USA) was used to navigate the microscope. Image capture parameters were set to pixel binning 1, the space between each z-layer to 0.6 µm and the z-depth of each capture was on average 25 layers per picture. A total number of 5 to 10 confocal image stacks were captured per compound and per experiment. A total amount of at least 120 cells were analyzed for all compounds. The PLA signals were analyzed using the open source image analysis program Image J (NIH, Bethseda, USA) and the amount of PLA signal per cell was calculated. Two persons analyzed the images independently in a blind fashion.

### Image analysis

In order to quantify the PLA signal from the total z-depth of each cell confocal z-stacks was projected in z and identically processed regarding background subtraction. The PLA signal was then quantified blindly and automatically using a find maxima-based algorithm in ImageJ (plugin routine developed by Dr. E. Flaberg and Professor L. Szekely, KI and available upon request). The images for visualization of the results were identically processed regarding background subtraction.

### Immunoblotting

Whole cell extracts were prepared and lysed using RIPA-buffer. Western blot analysis was performed as previously described [54]. In brief, membranes were probed with a mouse anti-MYCN antibody (B8.4.B, St Cruz Bio), MAX antibody (C-2, St Cruz Bio) and an HRP-conjugated anti-mouse antibody (DAKO) was used as secondary antibody. The membranes were developed using enhanced chemiluminescence substrate (ECL, Amersham). The membranes were subsequently re-probed with antibodies specific for GAPDH (6C5, St Cruz Bio) in order to confirm equal loading.

### Apoptosis detection

Small molecule-induced apoptosis was scored by quantifying the sub-G0/G1 fraction of the cell cycle. Treated neuroblastoma cells, were fixed in 70% ice-cold methanol and stored at −20°C. Cells were stained with propidium iodide solution (5 µg/ml propidium iodide and 25 µg/ml RNase A in PBS) for 30 min at 37°C and analyzed in a FACScan flow cytometer (Becton Dickinson). Apoptotic cells were identified within the PI-stained population by virtue of exhibiting an apparent sub-diploid DNA content. Tet21N cells were pre-treated with doxycycline for 4 days to turn *MYCN* off or grown without doxycycline. Both cell sets were incubated with the respective compounds followed by FACS analysis.

### Crystal violet staining

Cell viability was assayed by crystal violet staining. Kelly cells treated for 48 h with the indicated compound or DMSO were fixed in 1% trichloroacetic acid (TCA) (Merck-Schuchardt) for 1 h in 4°C and stained with 0.04% crystal violet (Sigma) for 30 min at room temperature. Following washing of unbound stain in water, the bound crystal violet was dissolved in 1% SDS. The absorbance was measured at 570 nm using 650 nm as reference wavelength. The IC_50_ was determined after scaling the percentage of viable cells in log using a nonlinear regression with graph pad prism 6.

### Neuronal differentiation of NB cells

SK-N-BE(2) cells were grown for 15 days in 6 well-plates and treated twice per week with DMSO or compound, with and without NGF. Differentiation was assessed based on number and length of neuronal outgrowths using a phase contrast microscope (Axiovert 40 CFL, Zeiss) and the Axiovision Rel. 4.8 software.

### Oil Red O staining of lipids

SK-N-BE(2) cells grown on cover slips were fixed in 4% PFA, washed in PBS and then in 60% isopropanol before staining with filtered Oil Red O solution (2 mg/ml) in 60% isopropanol. Cells were washed briefly in distilled water and mounted onto glass slides using water-soluble mounting medium (Aqua Pertex, Histolab products AB). For visualization, bright field images were captured at 40X magnification using an Axiovert 40 CFL inverted fluorescence microscope (Zeiss) and Axiovision Rel. 4.8 software.

## Supporting Information

Figure S1 **Chemical structures of the compounds used in this study.**
(TIF)

Figure S2 **Circular Dichroism spectra for the bHLHZip domains of c-MYC and MYCN**. The spectra of both proteins are similar to a model spectrum of an α-helical protein with minima at 208 and 222 nm. Shown spectra are averaged from three individual measurements.
(TIF)

Figure S3 **BIAcore sensorgrams for compound binding to the bHLHZip of c-MYC and MYCN.** The solvent corrected and background subtracted sensorgrams of c-MYC are displayed in the left panel, while the corresponding sensorgrams for MYCN are displayed in the right panel. The concentrations used are indicated to the right. All data was plotted with the injection point at 0 s. The reported K_D_-values are an average of at least three independent measurements and at least two different immobilizations.
(TIF)

Figure S4 **Proximity ligation assay control experiments.** SMS-KCN69n cells were treated with DMSO for 6 hours. Cover slips were incubated as described in the PLA procedure except for incubation with primary antibody where the following controls were used as indicated: no primary antibody, MYCN antibody, MAX antibody, MYCN and FAS antibodies or MAX and GFP antibodies. DNA was stained with DAPI. Scale bar: 10 µM. Photographs are representative from three independent experiments.
(TIF)

Figure S5 **Cell death induction in cells with an inducible** ***MYCN*** **expression.** Tet21N cells were treated with 1 ug/ml of doxycycline for downregulation of MYCN expression for 96 hours followed by a 48 hour treatment with the respective small molecules. In cells with and without MYCN expression. A) Western blot analysis of MYCN and MAX expression with and without doxycycline treatment. GAPDH was used as loading control. One representative blot from three independent experiments is shown. B) Quantification of cell death by propidium iodide staining for sub G1 DNA content of Tet21N (Tet-OFF) cells with high or low MYCN protein levels after treatment with the respective small molecules at the indicated concentrations. Data represent the means of at least three independent experiments. Error bars indicate standard error.
(TIF)

Figure S6 **Effects of C-** ***m/z*** **232, #474 and #764 on neuronal differentiation of NB cells.** Morphological differentiation of SK-N-BE(2) cells in response to 15 days culture with 10058-F4 (60 µM) C-*m/z* 232 (70 µM), #474 (20 µM), #764 (20 µM) or DMSO. Phase contrast micrographs show representative pictures from one out of 3 to 5 independent experiments.
(TIF)

Table S1 **Comparison of secondary structure predictions for c-MYC_353-437_ and MYCN_380-464_ based on the CD spectra.**
(DOCX)
