# Supplementary figures and images for: Targeting of the MYCN Protein with Small Molecule c-MYC Inhibitors
Source: PLoS One. 2014 May 23;9(5):e97285. doi: 10.1371/journal.pone.0097285 (PMC4032254; doi:10.1371/journal.pone.0097285)

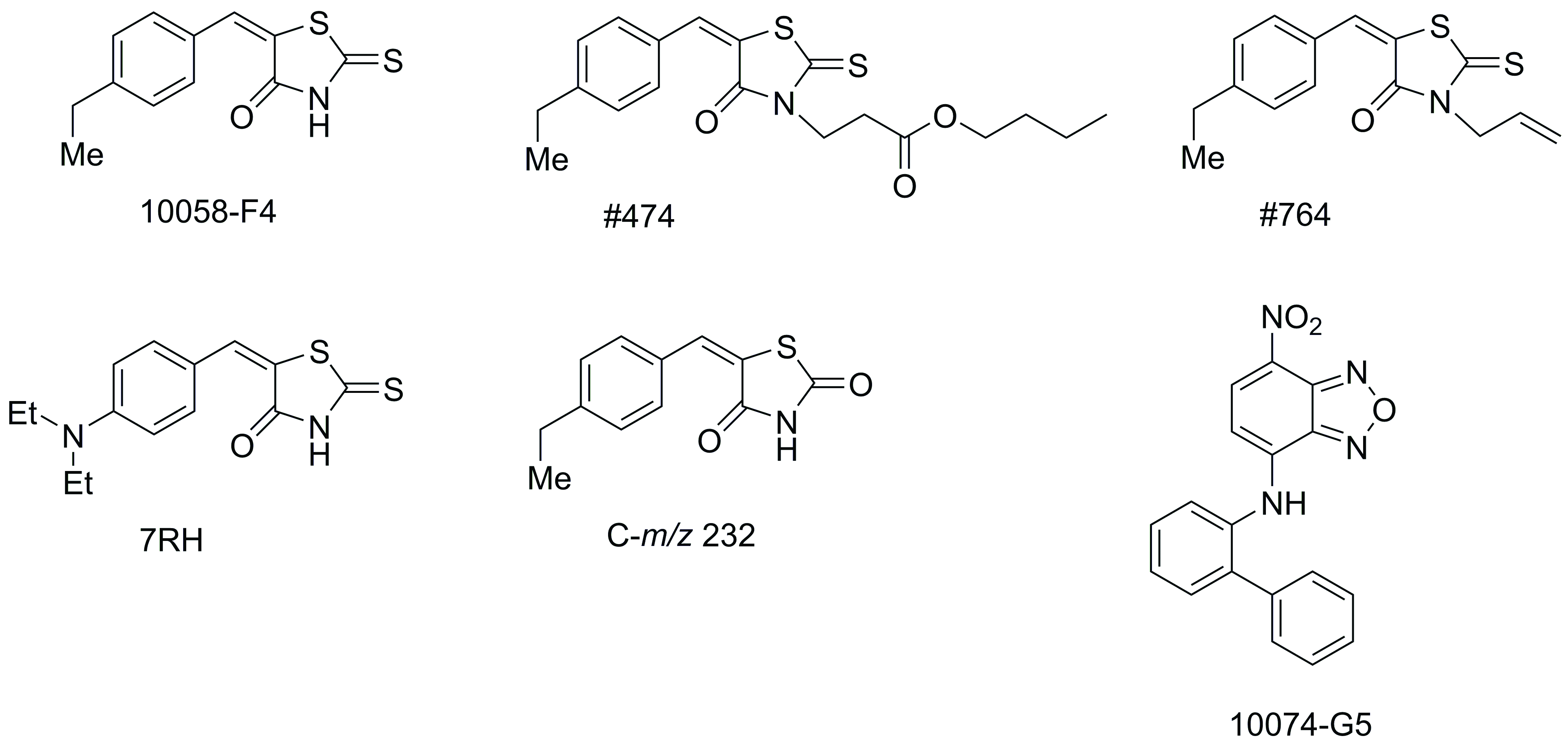

Supplement: Figure S1 — Chemical structures of the compounds used in this study. (TIF) [file pone.0097285.s001.tif]

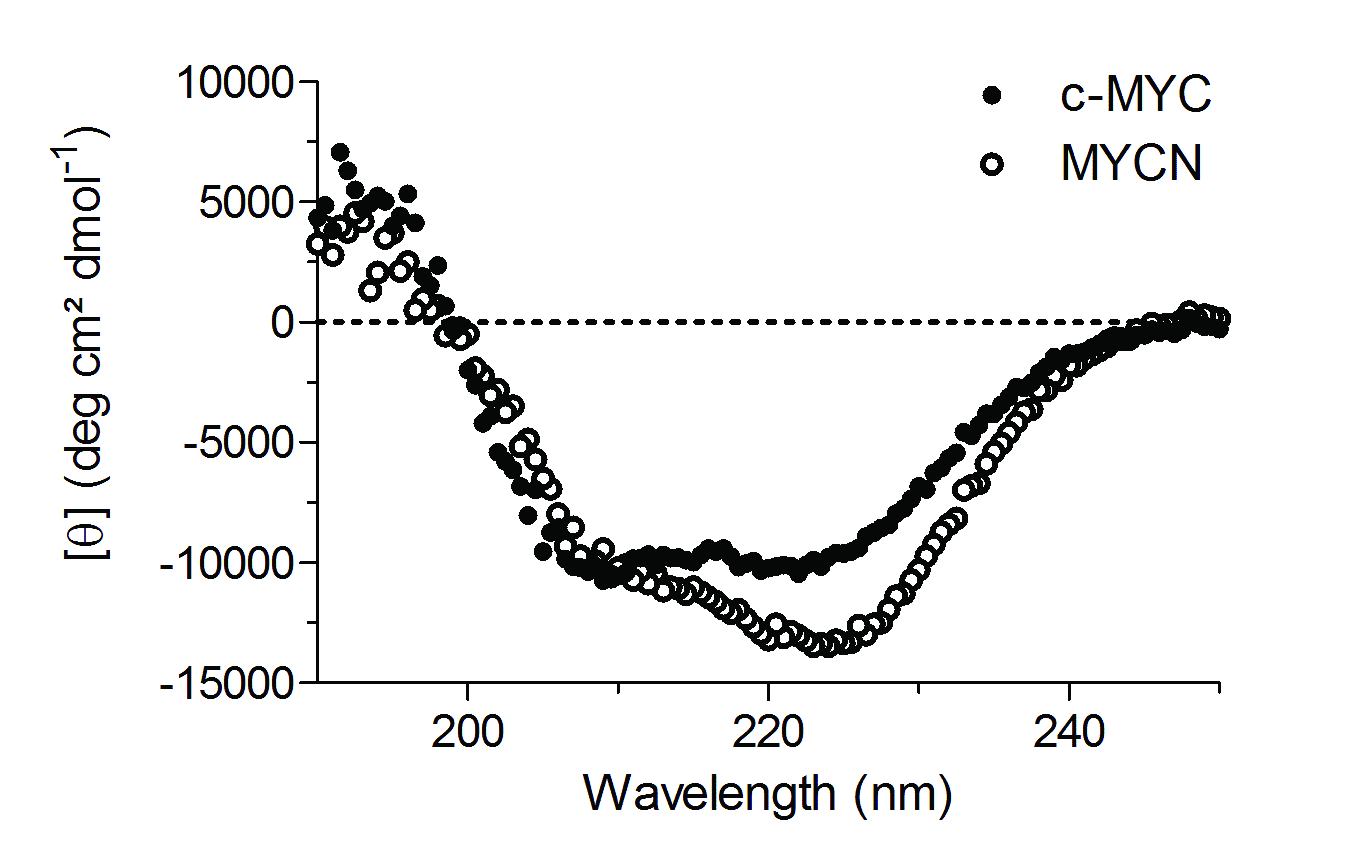

Supplement: Figure S2 — Circular Dichroism spectra for the bHLHZip domains of c-MYC and MYCN. The spectra of both proteins are similar to a model spectrum of an α-helical protein with minima at 208 and 222 nm. Shown spectra are averaged from three individual measurements. (TIF) [file pone.0097285.s002.tif]

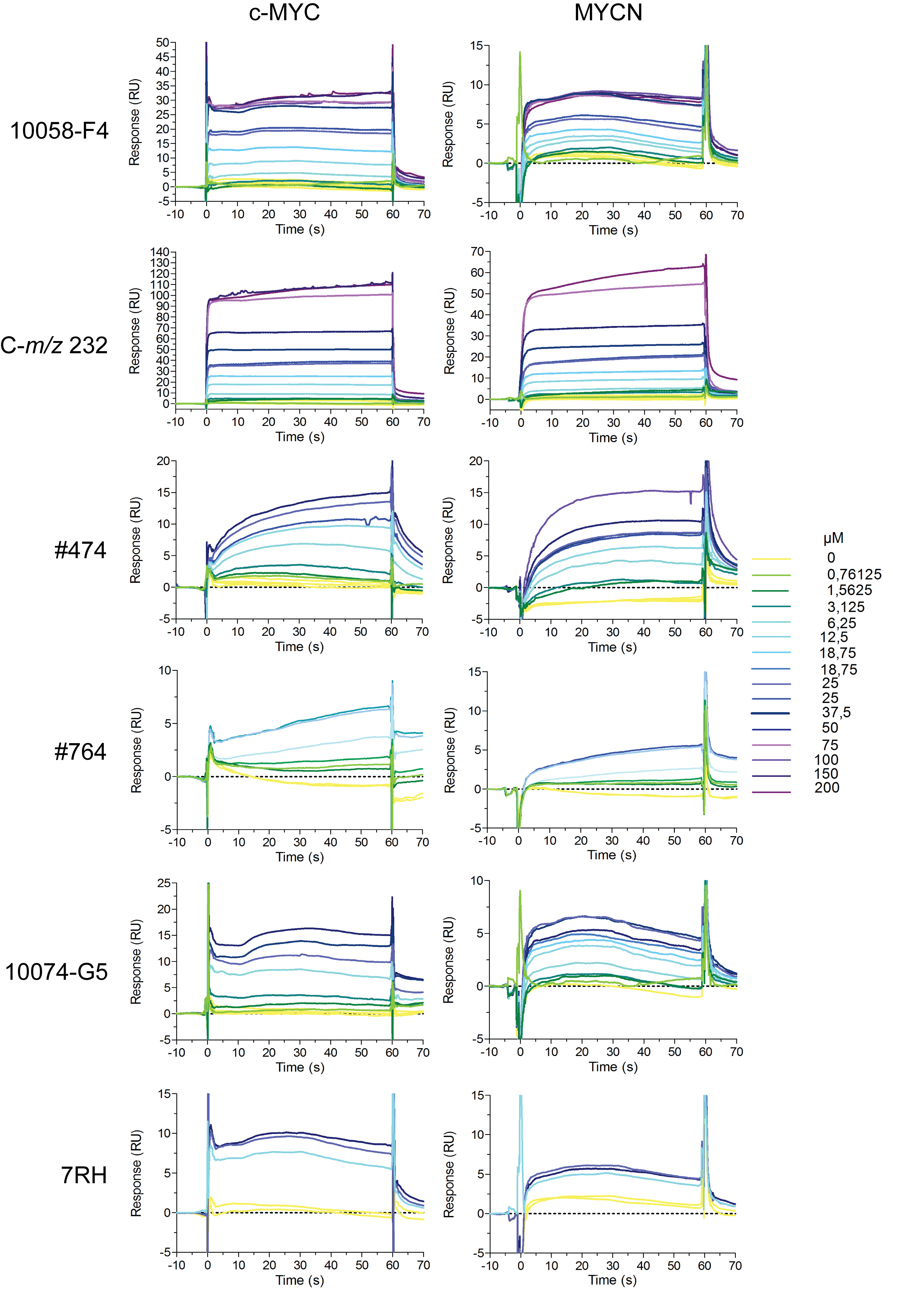

Supplement: Figure S3 — BIAcore sensorgrams for compound binding to the bHLHZip of c-MYC and MYCN. The solvent corrected and background subtracted sensorgrams of c-MYC are displayed in the left panel, while the corresponding sensorgrams for MYCN are displayed in the right panel. The concentrations used are indicated to the right. All data was plotted with the injection point at 0 s. The reported KD-values are an average of at least three independent measurements and at least two different immobilizations. (TIF) [file pone.0097285.s003.tif]

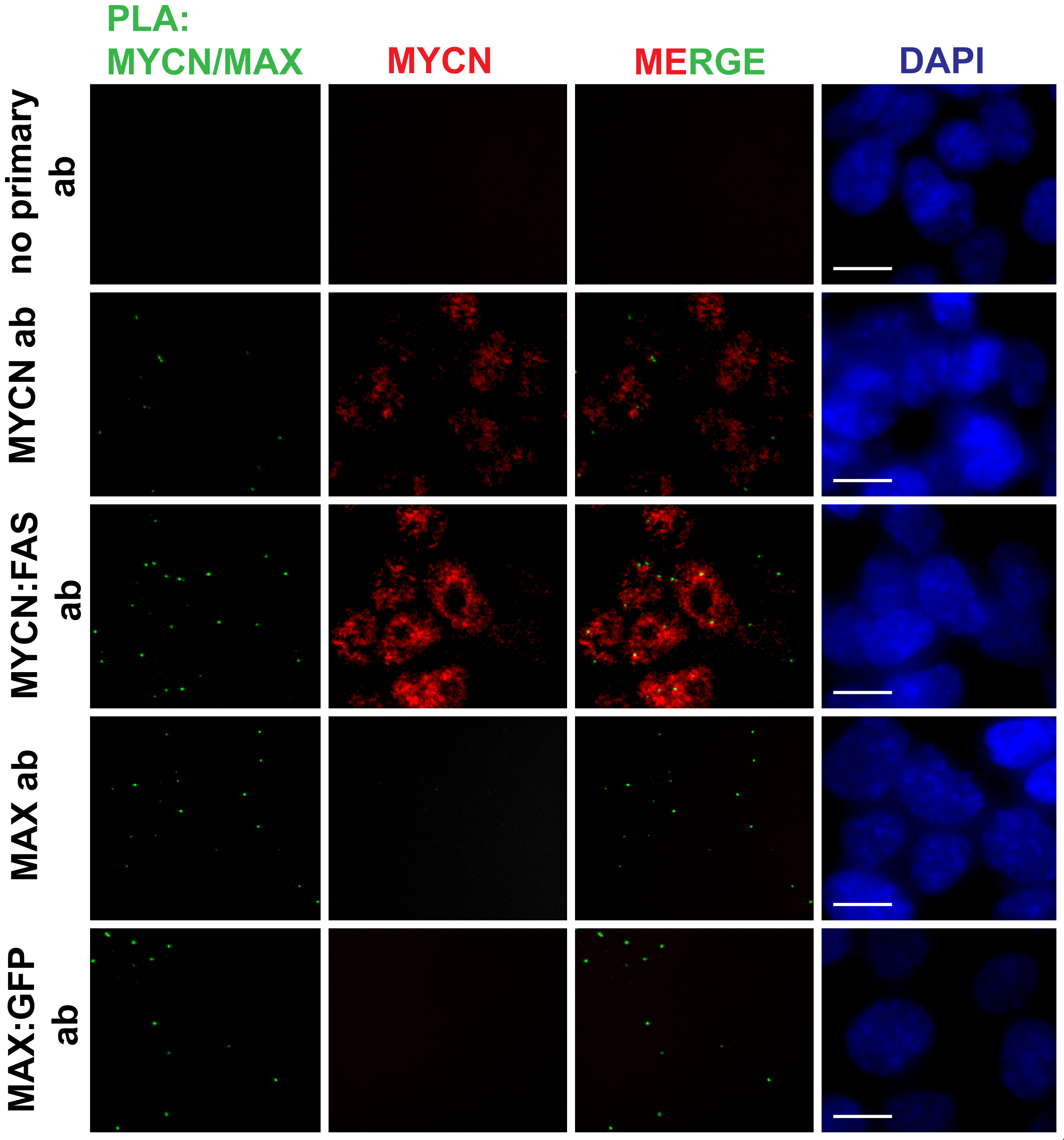

Supplement: Figure S4 — Proximity ligation assay control experiments. SMS-KCN69n cells were treated with DMSO for 6 hours. Cover slips were incubated as described in the PLA procedure except for incubation with primary antibody where the following controls were used as indicated: no primary antibody, MYCN antibody, MAX antibody, MYCN and FAS antibodies or MAX and GFP antibodies. DNA was stained with DAPI. Scale bar: 10 µM. Photographs are representative from three independent experiments. (TIF) [file pone.0097285.s004.tif]

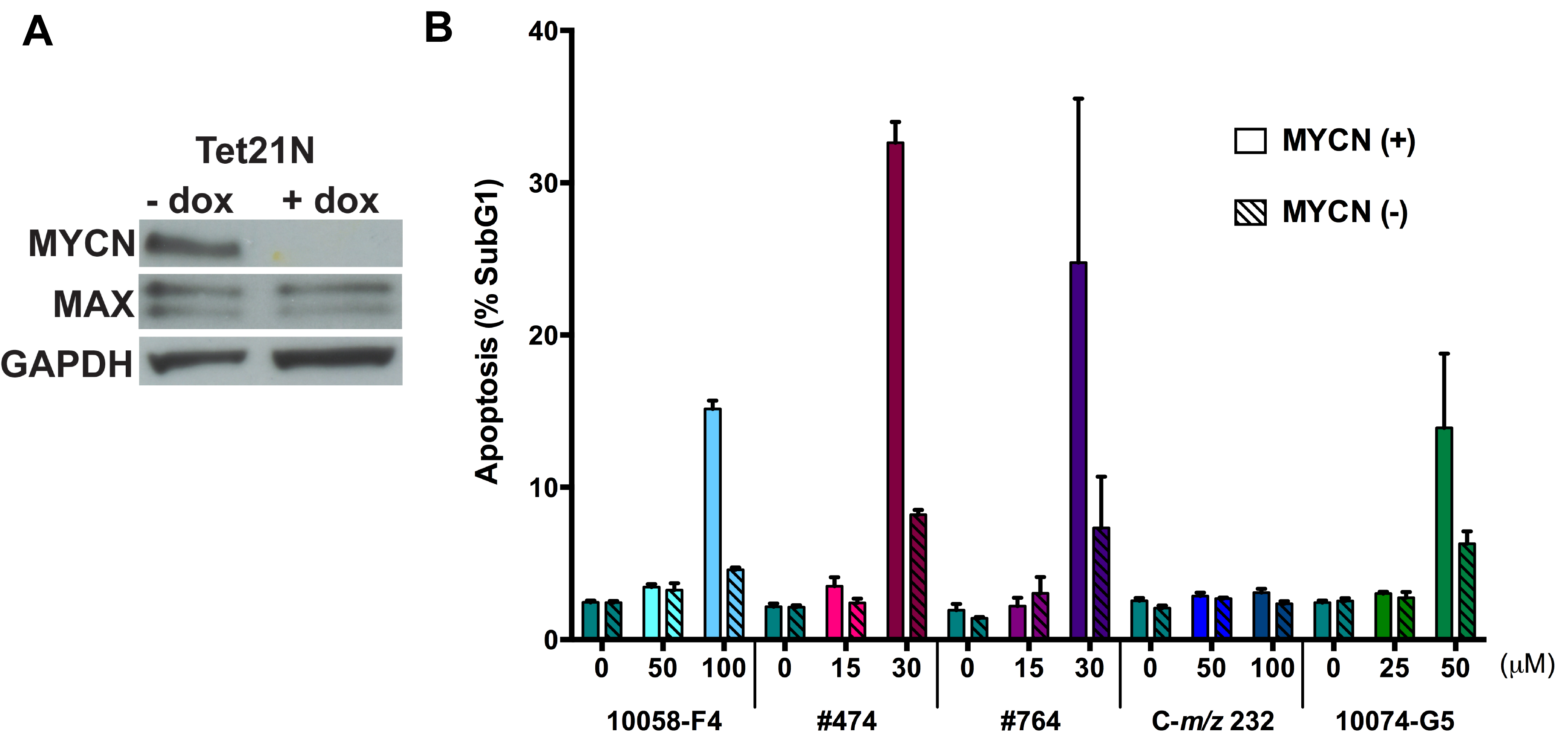

Supplement: Figure S5 — Cell death induction in cells with an inducible MYCN expression. Tet21N cells were treated with 1 ug/ml of doxycycline for downregulation of MYCN expression for 96 hours followed by a 48 hour treatment with the respective small molecules. In cells with and without MYCN expression. A) Western blot analysis of MYCN and MAX expression with and without doxycycline treatment. GAPDH was used as loading control. One representative blot from three independent experiments is shown. B) Quantification of cell death by propidium iodide staining for sub G1 DNA content of Tet21N (Tet-OFF) cells with high or low MYCN protein levels after treatment with the respective small molecules at the indicated concentrations. Data represent the means of at least three independent experiments. Error bars indicate standard error. (TIF) [file pone.0097285.s005.tif]

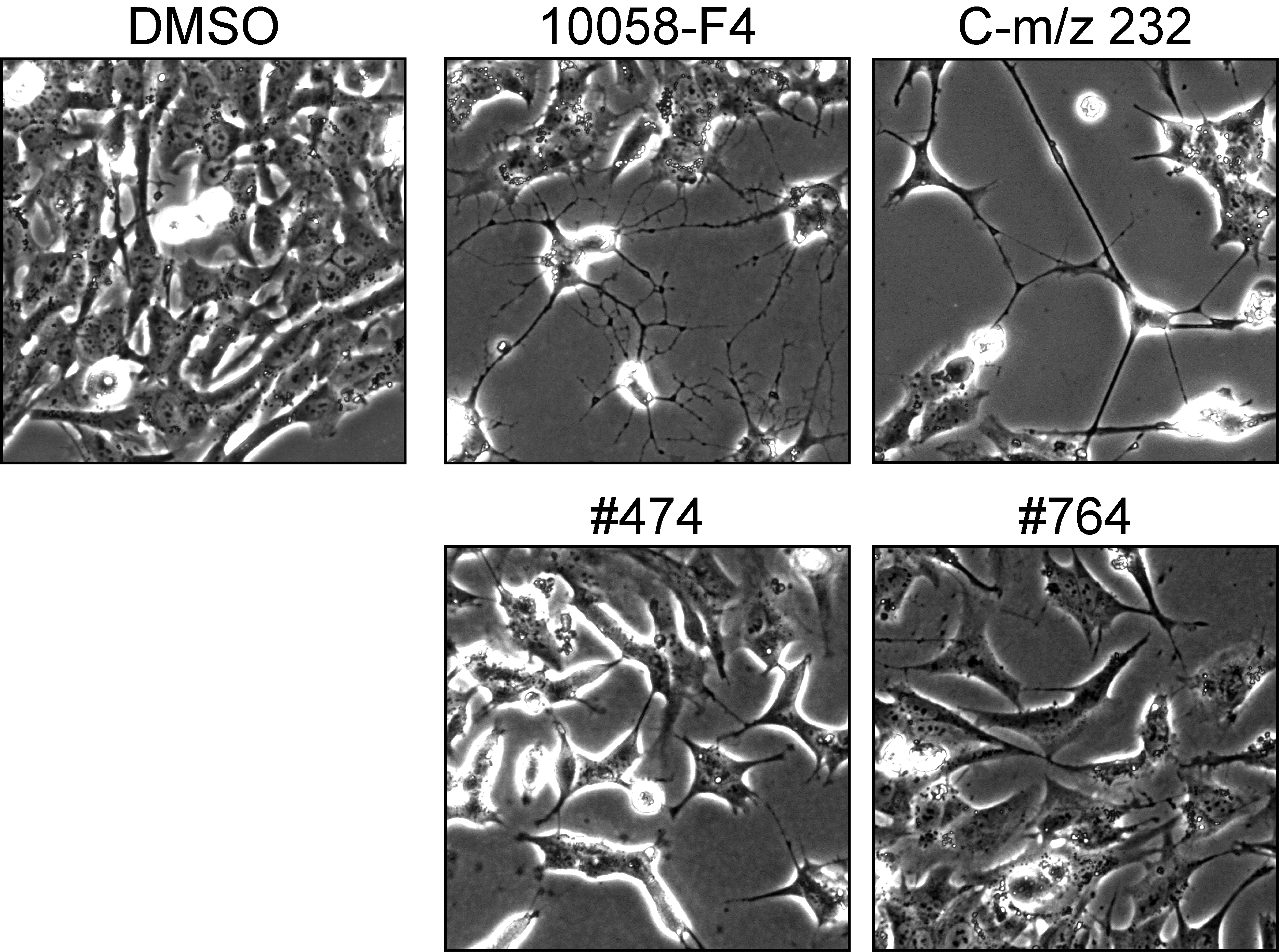

Supplement: Figure S6 — Effects of C- m/z 232, #474 and #764 on neuronal differentiation of NB cells. Morphological differentiation of SK-N-BE(2) cells in response to 15 days culture with 10058-F4 (60 µM) C-m/z 232 (70 µM), #474 (20 µM), #764 (20 µM) or DMSO. Phase contrast micrographs show representative pictures from one out of 3 to 5 independent experiments. (TIF) [file pone.0097285.s006.tif]
