# Supplementary material for: Targeting of the MYCN Protein with Small Molecule c-MYC Inhibitors
Source: PLoS One. 2014 May 23;9(5):e97285. doi: 10.1371/journal.pone.0097285 (PMC4032254; doi:10.1371/journal.pone.0097285)
Supplement: Table S1 — Comparison of secondary structure predictions for c-MYC353-437 and MYCN380-464 based on the CD spectra. (DOCX) [file pone.0097285.s007.docx]

|  | **Secondary structure (%)** | |  |
| --- | --- | --- | --- |
|  | **α-helix** | **β-sheet** | **Turn/unstructured** |
| c-MYC | 26.3 ± 3.7 | 15.9 ± 4.5 | 58.0 ± 3.5 |
| MYCN | 32.8 ± 4.7 | 12.7 ± 6.4 | 54.9 ± 1.64 |
